# Polymorphism in the *LASP1* gene promoter region alters cognitive functions of patients with schizophrenia

**DOI:** 10.1038/s41598-019-55414-1

**Published:** 2019-12-11

**Authors:** Chieh-Hsin Lin, Sheng Yang, Yu-Jhen Huang, Hsien-Yuan Lane

**Affiliations:** 1 Department of Psychiatry, Kaohsiung Chang Gung Memorial Hospital, Chang Gung University College of Medicine, Kaohsiung, Taiwan; 2 School of Medicine, Chang Gung University, Taoyuan, Taiwan; 3 Graduate Institute of Biomedical Sciences, China Medical University, Taichung, Taiwan; 4 Department of Psychiatry, China Medical University Hospital, Taichung, Taiwan; 5 Brain Disease Research Center, China Medical University Hospital, Taichung, Taiwan; 6 Department of Psychology, College of Medical and Health Sciences, Asia University, Taichung, Taiwan

**Keywords:** Disease genetics, Schizophrenia

## Abstract

Schizophrenia’s pathogenesis remains elusive. Cognitive dysfunction is the endophenotype and outcome predictor of schizophrenia. The LIM and SH3 domain protein (LASP1) protein, a component of CNS synapses and dendritic spines, has been related to the N-methyl-D-aspartate receptor (NMDAR) dysfunction hypothesis and schizophrenia. A single-nucleotide polymorphism (rs979607) in the *LASP1* gene promoter region has been also implicated in schizophrenia susceptibility. The aim of this study was to investigate the role of the *LASP1* rs979607 polymorphism in the cognitive functions of patients with schizophrenia. Two hundred and ninety-one Han Taiwanese patients with schizophrenia were recruited. Ten cognitive tests and two clinical rating scales were assessed. The scores of cognitive tests were standardized to T-scores. The genotyping of the *LASP1* rs979607 polymorphism was performed using TaqMan assay. Among the 291 patients, 85 were C/C homozygotes of rs979607, 141 C/T heterozygotes, and 65 T/T homozygotes, which fitted the Hardy-Weinberg equilibrium. After adjusting age, gender, and education with general linear model, the C/C homozygotes performed better than C/T heterozygotes in overall composite score (p = 0.023), Category Fluency test (representing processing speed and semantic memory) (p = 0.045), and Wechsler Memory Scale (WMS)-III backward Spatial Span test (p = 0.025), albeit without correction for multiple comparisons for the latter two individual tests. To the best of our knowledge, this is the first study suggesting that the genetic variation of *LASP1* may be associated with global cognitive function, category verbal fluency, and spatial working memory of patients with schizophrenia. The finding also lends support to the NMDAR dysfunction hypothesis of schizophrenia. More studies with longitudinal designs are warranted.

## Introduction

Dendritic spines are micrometer protrusion on neuronal dendrites containing the majority of excitatory synapses in human brain. During plasticity, dendritic spine undergoes structural changes which is primarily driven by dynamic remodeling of actin cytoskeleton^1^. Dendritic spine is crucial in both acquisition of new information (learning)^2^ and long-term information detention (memory formation)^3^, as well as cognitive functions^4^.

Schizophrenia is a complex disease that affects around 0.5%-1% of global population, and it brings about substantial societal, familial, and economical burdens. Clinical manifestations of schizophrenia include positive symptoms, negative symptoms, and cognitive impairments, with cognitive symptoms being an important influencer of patients’ long-term functional outcome^5–7^. Defects in dendritic spine morphology have been implicated in several neurodegenerative and neuropsychiatric diseases, including Alzheimer’s disease, bipolar disorder, and schizophrenia^8–11^. Decrease in dendritic spine density is observed in dorsolateral prefrontal cortex (DFPLC) of schizophrenia patients, compared to healthy control^12^.

LIM and SH3 domain protein 1 (LASP1) was initially cloned from a cDNA library of breast cancer metastases^13,14^. This protein contains an N-terminal LIM domain, which is composed of two sequential zinc-binding modules with a typical LIM motif, followed by tandem 35-residue nebulin-like repeats named R1 and R2, and by a C-terminal SRC homology region 3 (SH3) domain. The unique structure allows LASP1 to interact and bind with various proteins^15^. Its overexpression has been associated with multiple cancer aggressiveness including prostate carcinoma^16^, metastatic colorectal carcinoma^17^, ovarian cancer^18^ and nasopharyngeal carcinoma^19^.

LASP1 is also highly expressed in CNS, especially in cortex, cerebellum, and hippocampus, and is concentrated at synapses^20,21^. It was identified as one of actin-associated proteins in the post-synaptic densities (PSD)^20^. The two nebulin repeats region on LASP1 has been shown to interact and co-localize with F-actin^22^, one of the major cytoskeleton component, crucial in cell migration^23,24^, intracellular trafficking^25,26^, and maintaining dendritic spine structure and density^27,28^. LASP1 is also shown to participate in the stabilization of actin filaments bundles^29^. In cultured hippocampal neuronal differentiation, LASP1 is first observed in growth cones, followed by distribution throughout the dendrites^30^. Double labeling with excitatory synapses marker PSD-95 also showed LASP1 clustering at postsynaptic densities of dendritic spine^20^. This hints the possible role of LASP1 in dendritic spine development and morphology, and also in synaptic plasticity.

NMDA receptor (NMDAR) blockade is correlated with decreased spine density and dendritic length^31,32^. Furthermore, hypofunction of NMDAR is associated with schizophrenic symptoms and schizophrenia^33^. In mice treated with MK-801, an NMDAR antagonist, the level of LASP1 was down-regulated in the brain slices, especially in the frontal cortex region, suggesting its potential relation to NMDAR’s hypofunction^34^. Case-control population study also showed that T allele of rs979607, a single nucleotide polymorphism (SNP) of the *LASP1* gene promoter region, was associated with schizophrenia susceptibility in Korean population^34^. However, whether it would be related with cognitive function of patients with schizophrenia has not yet been investigated.

Based on the aforementioned findings, the actin-binding protein LASP1 has a potential role in regulating dendritic spine growth and morphology. Alterations in dendritic spine have been implicated in the development of schizophrenia and cognitive deficits^35,36^. Therefore, this study sought to test the influence of the *LASP1* polymorphism (*rs979607*) on cognitive function of patients with schizophrenia.

## Methods

The study was approved by institutional review board of China Medical University Hospital, Taiwan and conducted in accordance with the current revision of the Declaration of Helsinki. All participants were recruited from the chronic wards of China Medical University Hospital, Taiwan. The patients were included if they were (1) aged between 18 and 65; (2) diagnosed as schizophrenia by research psychiatrists using the Structured Clinical Interview for DSM-IV (1994); (3) with laboratory assessments (including blood routine, biochemical tests) within normal range; (4) with sufficient education to communicate effectively and complete the assessments of the study; and (5) under stable dosages of antipsychotics treatment for at least two months before test enrollment. Patients were excluded when they (1) presented with other comorbid psychiatric disorders, substance use disorder, mental retardation; (2) had other existing physical or neurological illnesses; and (3) failed to cooperate with the study. A total of 291 Han Taiwanese schizophrenic patients were recruited after they agreed to participate in the study and provided written informed consent after complete description of the study.

Patients’ clinical manifestations were measured by Positive and Negative Syndrome Scale (PANSS)^37^, Scale for Assessment of Negative Symptoms (SANS)^38,39^. All the participants received clinical ratings by trained and experienced research psychiatrist.

Interrater reliability was analyzed with the ANOVA test. Only raters achieving intra-class correlation coefficients of 0.90 or higher during pre-study training were allowed to rate the study patients.

Patients’ cognitive functions were measured by a battery, which included 7 domains: (1) speed of processing, which is composed of 3 subtests: Category Fluency^40–42^, Trail Making A^43^, and Wechsler Adult Intelligence Scale (WAIS)-III Digit Symbol-Coding^44^; (2) Continuous Performance Test for sustained attention^45,46^; (3) working memory, which is composed of verbal subtest (backward digit span)^47^ and nonverbal subtest (Wechsler Memory Scale [WMS]-III-Spatial Span, backward)^48^; (4) verbal learning and memory (WMS-III, word listing) (Wechsler, 1997)^48^; (5) visual learning and memory (WMS-III, visual reproduction)^48^; (6) reasoning and problem solving (Wechsler Intelligence Scale for Children [WISC]-III, Maze)^49^, and (7) Mayer–Salovey–Caruso Emotional Intelligence Test (MSCEIT)^50^ used for social cognition scaling, as recommended by the committee of US National Institute of Mental Health Measurement and Treatment Research to Improve Cognition in Schizophrenia (MATRICS) as the solitary measure of social cognition in schizophrenia^51^. The Chinese version of MSCEIT tasks was translated from English to Mandarin Chinese with satisfactory reliability, validity^52^, and applicability^53^. The cognitive battery had been successfully applied in previous clinical trials on schizophrenia^54,55^.

To analyze the rs979607 polymorphism of *LASP1*, the Master Pure DNA Purification Kit for Blood Version II (EPICENTRE, Madison, Wisconsin, USA) was used to isolate DNA from patients’ blood. We used ND-1000 UV-Vis spectrophotometer (Thermo Fisher Scientific Inc.) to determine the concentration of DNA by absorbance at 260 nM. DNA was then diluted to a concentration of 50 ng/ul and underwent PCR amplification reaction. For all SNP genotyping, the Taqman SNP genotyping assay (ABI: Applied Biosystems Inc., Foster City, CA, USA) was used. DNA samples of known genotypes were used in every reaction as positive controls.

The PCR reaction was conducted in the 10 μl reaction volume, containing 0.4 μl DNA sample, 5 μl PCR master mix, and 0.25 μl primer pairs and probes. A pre-incubation at 95 °C for 10 min was used to activate the Hot-Start DNA polymerase and denature DNA and was followed by 40 amplification cycles of 92 °C denaturation for 15 Sec; and 60 °C for 60 Sec. The probe fluorescence signal detection was performed using the ABI Prism 7500 Real-Time PCR System.

### Statistical analysis

Statistical Package for the Social Sciences (SPSS) version 17 (IBM Inc.) was used to analyze the data. The scores of the cognitive tests were all standardized to T-score. Among the genetic groups, the demographic and clinical symptoms groups differences were tested by Chi-Square test, Analysis of Variance (ANOVA) or Kruskal-Wallis test, according to the normality of the data. One-way ANOVA was performed to examine the effects of different SNP genotypes on cognitive functions. In addition, general linear models (GLM) was applied to further investigate the genetic effect of *LASP1* on cognitive functions while controlling for patients’ gender, age, and duration of education. Finally, to test the Hardy-Weinberg equilibrium of the genotype counts, a Chi-Square goodness-of-fit test was employed.

## Results

Two hundred and ninety-one patients with stable, chronic schizophrenia were enrolled and successfully genotyped for the *LAPS1* rs979607 SNP. The mean age of the patients was 38.2 ± 9.4 years old. The mean education level was 10.9 ± 2.4 years. The mean age of illness onset was 23.1 ± 6.7 years old, and mean illness duration lasted 188.4 ± 252.5 months. The mean dosage of antipsychotics used, shown as the equivalent to chlorpromazine, was 531.0 ± 497.3 mg per day. No significant difference between genotypes of LASP1 rs979607 and demographic data was observed (Table 1).

**Table 1:** Demographics of schizophrenia patients with three *LASP1* genotypes.

	C/C	C/T	T/T	P value
Gender, male/female	57/28	80/61	35/30	0.19
Age, year, mean (SD)	38.4 (9.8)	38.1 (9.5)	38.2 (8.4)	0.98
Education, year, mean (SD)	10.7 (2.5)	10.9 (2.4)	11.0 (2.3)	0.75
Age at illness onset, year, mean (SD)	23.2 (7.2)	22.9 (6.5)	23.5 (6.4)	0.77
Illness duration, month, mean (SD)	178.4 (102.7)	203.4 (348.1)	169.0 (95.0)	0.93
Chlorpromazine equivalent dose of antipsychotics, mg, mean (SD)	498.0 (285.3)	533.8 (600.4)	567.8 (471.4)	0.89

Chi-square test for categorical variables (gender); and Kruskal-Wallis test for continuous variables, according to the normality of data.

The mean scores of PANSS total, PANSS-positive subscale, PANSS-negative subscale, and SANS 20-total were 85.2 ± 13.3, 20.0 ± 4.6, 23.9 ± 5.1, 51.1 ± 15.9, respectively. No significant differences were observed in PANSS total score, PANSS-positive subscale, PANSS-negative subscale, and SANS 20-total score among three *LASP1* rs979607 genotypes (Table 2).

**Table 2:** Clinical symptoms of schizophrenia patients with three *LASP1* genotypes.

	C/C	C/T	T/T	P value
PANSS total	82.7 (12.2)	86.5 (13.9)	85.6 (13.2)	0.14
PANSS-positive subscale	19.9 (4.5)	20.1 (4.5)	19.6 (4.9)	0.74
PANSS-negative subscale	22.9 (4.5)	24.3 (5.4)	24.5 (5.2)	0.053
SANS total	48.3 (15.9)	51.6 (16.2)	53.8 (14.8)	0.097

^1^Data presented by mean (SD).

^2^According to the normality of data, ANOVA was used for SANS total and Kruskal-Wallis test for other categories.

^3^PANSS, Positive and Negative Syndrome Scale.

^4^SANS, Scale for the Assessment of Negative Symptoms (20 items).

Of the 291 patients, all were assayed using the Taqman SNP genotyping method: 85 showed the C/C genotype of the *LASP1* rs979607 SNP, 141 had the C/T genotype, and 65 had the T/T genotype. This genotype distribution was in equilibrium with the Hardy-Weinberg law (p = 0.653). Regarding the allele distribution, the minor allele frequency of rs979607 in our study (T allele: 46.6%) was similar to that of Han Chinese populations (43.0%) from HapMap database (www.hapmap.org). However, our study yielded a different minor allele (T) from that (C) of the Korean patients (Table 3). Three positive controls (C/C, C/T and T/T) were added while analyzing the *LASP1* rs979607 genotype, and the results of the positive control genotyping were in line with the expected types. Therefore, the genotyping error rate could be regarded as 0.

**Table 3:** *LASP1* allele distribution among Han Taiwanese schizophrenia patients (the present study), Korean schizophrenia patients, and general Han Chinese population.

	Han Taiwanese Schizophrenia patients (the present study)	Korean schizophrenia patients^34^	Han Chinese (HapMap database, www.hapmap.org)
C/C	0.292	0.234	0.326
C/T	0.485	0.502	0.488
T/T	0.223	0.264	0.186
MAF	0.466 (T allele)	0.485 (C allele)	(T allele)

MAF: Minor allele frequency.

We further investigated the genetic effect of *LASP1* on cognitive functions (Table 4). Cognitive functions among three genotypic groups of *LASP1* rs979607 failed to reach significant difference. However, schizophrenic patients with C/C homozygotes showed an insignificant trend of better performance in overall composite score (p = 0.070), Category Fluency (p = 0.089), and WMS-III Spatial Span (representing non-verbal working memory) (p = 0.079).

**Table 4:** Cognitive function in patients with different *LASP1* genotypes.

	C/C	C/T	T/T	p value
Overall composite score	51.0 (5.9)	49.2 (6.2)	50.4 (5.6)	0.070
Speed of processing	51.0 (8.0)	49.5 (7.5)	49.7 (7.1)	0.33
Category Fluency	51.5 (10.9)	48.7 (9.9)	50.9 (8.8)	0.089
Trail Making A	50.4 (11.1)	50.3 (9.7)	48.8 (9.2)	0.57
Digit Symbol-Coding, WAIS-III	51.2 (10.1)	49.6 (10.2)	49.3 (9.4)	0.40
Attention: CPT	50.8 (10.5)	49.2 (10.0)	50.7 (9.4)	0.41
Working memory	50.9 (8.8)	49.0 (9.5)	50.9 (7.7)	0.17
Verbal backward (digit span)	50.9 (10.1)	49.4 (10.3)	50.1 (9.2)	0.59
Non-verbal backward (WMS-III spatial span)	52.0 (11.1)	49.0 (9.6)	49.6 (9.0)	0.079
Verbal learning and memory (WMS-III word listing)	50.9 (8.5)	49.0 (10.6)	50.9 (10.4)	0.28
Visual learning and memory (WMS-III visual reproduction)	51.1 (11.0)	48.9 (9.0)	51.0 (10.6)	0.18
Reasoning and problem solving (WISC-III maze)	50.7 (10.2)	49.1 (9.8)	51.0 (10.2)	0.36
Social cognition (MSCEIT)	51.3 (9.8)	49.4 (10.4)	49.7 (7.1)	0.38

^1^All data standardized to T scores and presented by mean (SD).

^2^All data were analyzed by ANOVA.

^3^verall composite score: an overall composite T scores including all seven domains was calculated by standardizing the sum of T scores.

^4^Speed of processing: an overall composite T score including all 3 domains (Category fluency, Trail marking A, WAIS-III Digit symbol-coding) was calculated by standardizing the sum of T scores.

^5^Working memory: an overall composite T score including 2 domains (Backward digit span, WMS-III, Spatial span) was calculated by standardizing the sum of T scores.

^6^WAIS-III, Wechsler adult intelligence scale, 3^rd^ version.

^7^WMS-III, Wechsler memory scale, 3^rd^ version.

^8^WISC-III, Wechsler intelligence scale for children, 3^rd^ version.

^9^MSCEIT, Mayer-Salovey-Caruso emotional intelligence test.

Next, we applied general linear model (GLM) to adjust age, gender, and education. Schizophrenic patients with *LASP1* rs979607 C/C homozygotes performed significantly better than C/T heterozygotes in overall composite score (p = 0.023) (Table 5). At this first step, there was no need for multiple comparisons due to only one analysis.

**Table 5:** Analysis of the effects of *LASP1* genetic polymorphism on cognitive functions in schizophrenic patients by general linear model

	*LASP1* rs979607	C/C vs C/T	C/C vs T/T	C/T vs T/T
Overall composite score	Mean difference (SD)	1.8 (0.8)	0.6 (0.9)	−1.2 (0.9)
	p value, (95% CI)	**0.023**, (0.2, 3.3)	0.53, (−1.3, 2.4)	0.16, (−2.9, 0.5)
Speed of processing	Mean difference (SD)	1.5 (1.0)	1.4 (1.2)	−0.1 (1.1)
	p value, (95% CI)	0.13, (−0.5, 3.5)	0.26, (−1.0, 3.8)	0.89, (−2.3, 2.0)
Category Fluency	Mean difference (SD)	2.7 (1.4)	0.5 (1.6)	−2.3 (1.5)
	p value, (95% CI)	**0.045**, (0.1, 5.4)	0.76, (−2.7, 3,7)	0.13, (−5.2, 0.7)
Trail Making A	Mean difference (SD)	0.1 (1.4)	1.5 (1.6)	1.4 (1.5)
	p value, (95% CI)	0.96, (−2.6, 2.8)	0.36, (−1.7, 4.7)	0.34, (−1.5, 4.4)
Digit Symbol-Coding, WAIS-III	Mean difference (SD)	1.8 (1.3)	2.2 (1.6)	0.4 (1.4)
	p value, (95% CI)	0.17, (−0.8, 4.4)	0.17, (−0.9, 5.3)	0.80, (−2.4, 3.2)
Attention: CPT	Mean difference (SD)	1.4 (1.4)	−0.2 (1.6)	−1.5 (1.5)
	p value, (95% CI)	0.31, (−1.3, 4.1)	0.92, (−3.4, 3.0)	0.30, (−4.5, 1.4)
Working memory	Mean difference (SD)	2.0 (1.1)	1.4 (1.3)	−0.7 (1.2)
	p value, (95% CI)	0.059, (−0.1, 4.1)	0.29, (−1.2, 3.9)	0.57, (−3.0, 1.6)
Verbal backward (digit span)	Mean difference (SD)	1.2 (1.3)	0.6 (1.6)	−0.7 (1.4)
	p value, (95% CI)	0.35, (−1.4, 3.8)	0.72, (−2.5, 3.7)	0.65, (−3.5, 2.2)
Non-verbal backward (WMS-III spatial span)	Mean difference (SD)	2.8 (1.3)	2.2 (1.5)	−0.7 (1.4)
	p value, (95% CI)	**0.025**, (0.4, 5.3)	0.15, (−0.8, 5.2)	0.63, (−3.3, 2.0)
Verbal learning and memory (WMS-III word listing)	Mean difference (SD)	2.3 (1.2)	0.6 (1.5)	−1.7 (1.4)
	p value, (95% CI)	0.066, (−0.2, 4.8)	0.68, (−2.3, 3.6)	0.22, (−4.3, 1.0)
Visual learning and memory (WMS-III visual reproduction)	Mean difference (SD)	2.5 (1.3)	0.5 (1.6)	−2.0 (1.5)
	p value, (95% CI)	0.063, (−0.1, 5.2)	0.74, (−2.7, 3.7)	0.18, (−4.9, 0.9)
Reasoning and problem solving (WISC-III maze)	Mean difference (SD)	1.2 (1.3)	−0.8 (1.5)	−2.0 (1.4)
	p value, (95% CI)	0.35, (−1.3, 3.7)	0.58, (−3.8, 2.2)	0.14, (−4.7, 0.7)
Social Cognition (MSCEIT)	Mean difference (SD)	1.6 (1.3)	1.2 (1.6)	−0.4 (1.4)
	p value, (95% CI)	0.23, (−1.0, 4.2)	0.44, (−1.9, 4.4)	0.79, (−3.2, 2.5)

^1^All data were standardized to T scores.

^2^95% CI = 95% confidence interval. P-value was based on the cognitive function between genetic groups multiple regression model analysis of by general linear model. Gender, age, and years of education were adjusted in the model.

After obtaining a positive finding, we then examined the significance of various cognitive domains. Schizophrenic patients with LASP1 rs979607 C/C homozygotes performed significantly better than C/T heterozygotes in category fluency (p = 0.045), and WMS-III-Spatial Span (p = 0.025) (Table 5). In addition, the C/C homozygotes also showed an insignificant trend to perform better than C/T heterozygotes in overall composite score, category fluency, and WMS-III-Spatial Span (Table 5). At this secondary step, correction of multiple comparisons could be imposed. In this case, none of the cognitive domains reached statistical significance.

## Discussion

The results of this study showed that schizophrenic patients with the C/C genotype of *LASP1* rs979607 performed better than those with the C/T genotype in general cognitive function, category verbal fluency, and non-verbal (spatial) working memory **(**Table 5**)**. Since LASP1 protein has been related to the NMDA hypofunction theory of schizophrenia^34^ and NMDAR-related neurotransmission has been associated with cognitive function in schizophrenia patients^56^, it is reasonable that LASP1 also plays a role in the modulation of cognitive function.

Schizophrenia patients with homozygote alleles (particularly the C/C group) performed better than heterozygotes (C/T group) in some cognitive tests. The hypothesis named molecular heterosis implies that heterozygote subjects result in a greater or lesser impact on specific traits compared with homozygotes for a specific genetic polymorphism. Examples include smoking and cognitive functions in schizophrenic patients^57–59^. Our findings appeared in accordance with molecular heterosis.

Schizophrenia patients with C/C genotype performed better than C/T heterozygotes on category fluency in this study. Deficits in category fluency have been observed in schizophrenia patients^60,61^ and also in dementia patients^62,63^. Category fluency is reflective of speed of processing^64,65^ and semantic memory^66^. NMDARs specifically in CA3 pyramidal cells regulate speed of processing^67^, and NMDAR modulations (both agonism and antagonism) alter semantic memory^68,69^. In accordance, the current study suggests that *LASP1* genetic polymorphism may be related with different category fluency. More studies are needed to elucidate the mechanism of LASP1’s influence on speed of processing and semantic memory.

Spatial working memory varied with *LASP1* rs979607 polymorphisms of schizophrenia patients in this study. Deficit in spatial working memory is considered one of the core neurocognitive impairments and an endophenotype of schizophrenia^70–72^. NMDARs, specifically NR2B-containing NMDARs, are vital for spatial working memory^73,74^. Of note, functional loss of NMDARs in the dentate gyrus impairs spatial working memory but spares spatial reference memory^75^. Therefore, it is expectable that LASP1 may be involved in spatial working memory.

The limitations of this study included the following: First, schizophrenia is a heterogeneous disease, in which many factors contribute to its development and presentation. A SNP might only have limited influence on the cognitive function of schizophrenia. More studies are warranted to explore the potential roles of other markers. Second, the sample size was modest. Third, only Han Taiwanese patients were enrolled. Whether the findings could be extrapolated to other ethnicity populations remain unclear. Fourth, no healthy individuals were included in this study for comparison. Fifth, the study was cross-sectional, without longitudinal follow-up. In addition, whether the finding could be observed even during the prodromal phase of the illness also deserves research.

To the best of our knowledge, this is the first study describing genetic polymorphisms of *LASP1* may have impact on cognitive functions in patients with schizophrenia. The finding also lends support to the NMDA dysfunction theory of schizophrenia. Further studies with larger sample sizes of both schizophrenia patients and controls, various ethnicities, and longitudinal designs are needed to clarify the role of *LASP1* in schizophrenia and cognitive functions.
